# Supplementary material for: Modeling glioblastoma heterogeneity as a dynamic network of cell states
Source: Mol Syst Biol. 2021 Sep 16;17(9):e10105. doi: 10.15252/msb.202010105 (PMC8444284; doi:10.15252/msb.202010105)
Supplement: Supplementary file 6 — Source Data for Figure 5 [file MSB-17-e10105-s004.zip › Figure5A_sourcedata/GSEA_3071/hallmarks_stateI.GseaPreranked.1623742531165/HALLMARK_KRAS_SIGNALING_DN.html]

Details for gene set HALLMARK\_KRAS\_SIGNALING\_DN[GSEA]

|  || Dataset | state13071 |
| Phenotype | NoPhenotypeAvailable |
| Upregulated in class | na\_neg |
| GeneSet | HALLMARK\_KRAS\_SIGNALING\_DN |
| Enrichment Score (ES) | -0.4357664 |
| Normalized Enrichment Score (NES) | -1.6114818 |
| Nominal p-value | 0.04248366 |
| FDR q-value | 0.062388584 |
| FWER p-Value | 0.516 |
Table: GSEA Results Summary

  

Fig 1: Enrichment plot: HALLMARK\_KRAS\_SIGNALING\_DN      
 Profile of the Running ES Score & Positions of GeneSet Members on the Rank Ordered List

  

| PROBE | GENE SYMBOL | GENE\_TITLE | RANK IN GENE LIST | RANK METRIC SCORE | RUNNING ES | CORE ENRICHMENT || 1 | GPRC5C |  |  | 42 | 1.413 | 0.0658 | No |
| 2 | LFNG |  |  | 228 | 0.772 | 0.0553 | No |
| 3 | TENM2 |  |  | 309 | 0.664 | 0.0690 | No |
| 4 | IFI44L |  |  | 422 | 0.574 | 0.0685 | No |
| 5 | COPZ2 |  |  | 650 | 0.432 | 0.0273 | No |
| 6 | CAMK1D |  |  | 708 | 0.408 | 0.0334 | No |
| 7 | MX1 |  |  | 1130 | 0.302 | -0.0706 | No |
| 8 | CLSTN3 |  |  | 1805 | -0.294 | -0.2477 | No |
| 9 | SNN |  |  | 1986 | -0.316 | -0.2818 | No |
| 10 | YPEL1 |  |  | 2254 | -0.364 | -0.3383 | No |
| 11 | SLC25A23 |  |  | 2307 | -0.374 | -0.3326 | No |
| 12 | PTPRJ |  |  | 2312 | -0.374 | -0.3132 | No |
| 13 | BTG2 |  |  | 2318 | -0.375 | -0.2939 | No |
| 14 | DCC |  |  | 2643 | -0.451 | -0.3620 | No |
| 15 | CNTFR |  |  | 2697 | -0.471 | -0.3512 | No |
| 16 | CELSR2 |  |  | 2993 | -0.597 | -0.4029 | Yes |
| 17 | EDN1 |  |  | 3079 | -0.660 | -0.3909 | Yes |
| 18 | GAMT |  |  | 3141 | -0.723 | -0.3685 | Yes |
| 19 | BARD1 |  |  | 3201 | -0.783 | -0.3423 | Yes |
| 20 | TGFB2 |  |  | 3251 | -0.852 | -0.3094 | Yes |
| 21 | TFAP2B |  |  | 3274 | -0.886 | -0.2669 | Yes |
| 22 | KCNQ2 |  |  | 3383 | -1.142 | -0.2349 | Yes |
| 23 | KLHDC8A |  |  | 3438 | -1.433 | -0.1714 | Yes |
| 24 | IGFBP2 |  |  | 3509 | -3.484 | 0.0006 | Yes |
Table: GSEA details [plain text format]

  

Fig 2: HALLMARK\_KRAS\_SIGNALING\_DN: Random ES distribution      
 Gene set null distribution of ES for **HALLMARK\_KRAS\_SIGNALING\_DN**
